# Pathological Fracture of the Proximal Femur in Osteosarcoma: Need for Early Radical Surgery?

**DOI:** 10.5402/2012/512389

**Published:** 2012-03-18

**Authors:** C. R. Chandrasekar, R. J. Grimer, S. R. Carter, R. M. Tillman, A. Abudu, L. M. Jeys, W. G. H. Cheung, R. Sharma

**Affiliations:** ^1^Consultant Orthopaedic Surgeon, Department of Orthopaedics, Royal Liverpool and Broadgreen University Hospitals, NHS Trust, Prescot Street, Liverpool L7 8XP, UK; ^2^Consultant Orthopaedic Oncologist, ROH Bone Tumour Service, Royal Orthopaedic Hospital, Birmingham B31 2AP, UK; ^3^Department of Radiology, Royal Infirmary, Edinburgh EH16 4SA, UK; ^4^Department of Emergency Medicine, George Elliot Hospital, Nuneaton CV1O 7DJ, UK

## Abstract

Seventeen patients underwent treatment for a pathological fracture of the proximal femur due to osteosarcoma. Their age range was from 9 to 84 (mean age 42) with nine patients under the age of 40 and eight above the age of 40. Twelve patients had a fracture at diagnosis and five developed a fracture after the diagnosis. Seven patients had metastatic disease at diagnosis. Five patients were referred after internal fixation of the fracture prior to diagnosis. Chemotherapy was used when appropriate and eight patients then underwent limb salvage surgery, six had an amputation, and three had palliative treatment. The estimated five-year survival was 14%. These results are significantly worse than expected, and it proved impossible to identify any group who fared well. The high incidence of metastases both at diagnosis and subsequently suggests this group of patients are at very high risk. Review of multicentre data may suggest an optimum treatment for this patient group.

## 1. Introduction

Osteosarcoma is the commonest primary bone sarcoma arising with an incidence of approximately 3 per million population/year [8]. 70% of all osteosarcomas will arise around the knee, but 5-6% arise in the proximal femur. The incidence of pathological fracture in osteosarcoma is from 5% to 12%. There are many reports on osteosarcoma and pathological fracture, [1–16] most of these reports only dealing with patients without metastases at diagnosis. Pathological fracture can be the presenting feature or it can occur during treatment. Progressive loss of bone matrix, biopsy, and minor trauma are some of the reasons for the occurrence of a pathological fracture. The presence of a pathological fracture has been noted to be an adverse prognostic factor in osteosarcoma by some authors [9] but not by others [3, 5].

A pathological fracture of the proximal femur poses particular problems, because the fracture haematoma (which has to be considered to contain tumour cells) may either be intracapsular, thus contaminating the hip joint, or extracapsular when there is likely to be widespread contamination of surrounding tissues. Unlike the distal femur or proximal tibia where fracture haematomas are often contained by muscle (vastus intermedius and popliteus, resp.), there is no such limiting muscle in the proximal femur. Management is also difficult because it is difficult to immobilise the fracture either in plaster or with traction and continued movement at the fracture site during preoperative chemotherapy may increase the risk of wider local spread and possibly metastases. To our knowledge there is no published study addressing the outcome of pathological fractures of the proximal femur due to osteosarcoma, and the aim of the present study was to address this specific problem.

## 2. Patients and Methods

We retrospectively analysed data contained in a prospective tumour database. We included all patients who had a diagnosis of osteosarcoma of the proximal femur with a pathological fracture. We included intracapsular, intertrochanteric, and subtrochanteric fractures in the study.

**Figure 1:**
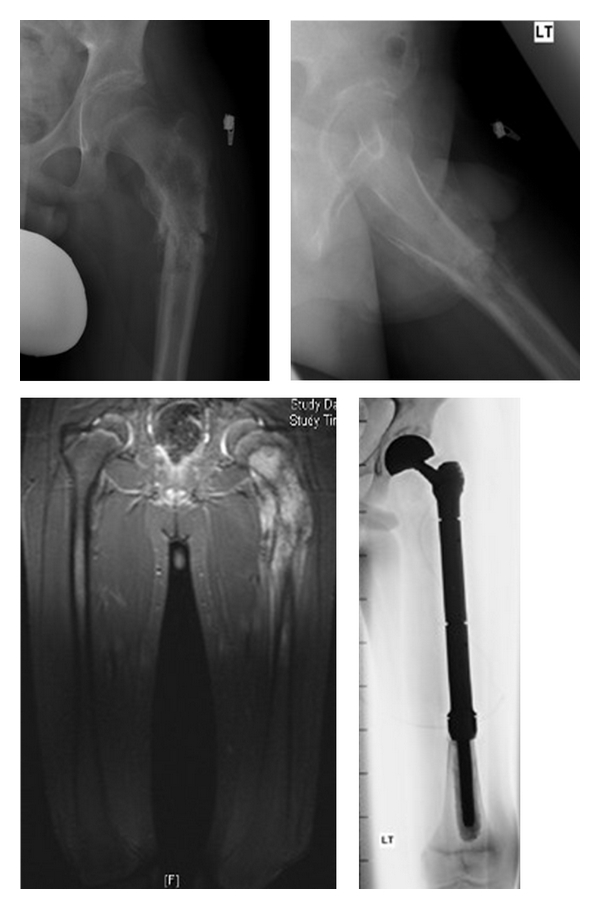
Preoperative radiographs, MRI scan of osteosarcoma of the proximal femur, and postoperative radiograph showing proximal femoral endoprosthesis.

Between 1978 and 2008, 1193 patients were diagnosed and treated for osteosarcoma in our unit. 54 patients (4.5%) had proximal femoral disease. Seventeen patients (31%) presented with a fracture or sustained a fracture of the proximal femur during the course of preoperative treatment. All had staging studies including bone scan, MRI scan of the proximal femur, and CT of the chest. (Figure 1) Tissue diagnoses were obtained in all the cases, usually by needle biopsy. Before operation, the fractures were immobilised by traction with the patient being on bed rest. The patients were offered preoperative chemotherapy according to the protocol in use at the time. In general, most patients under the age of 60 were offered neoadjuvant chemotherapy, and were then restaged prior to a decision being made about surgery. Patients over the age of 60 usually had surgery (if possible) without chemotherapy.

The decision as to the operation to be undertaken to control the local disease was based on the local extent of the tumour as seen on the imaging scans, the response to preoperative chemotherapy and patient preference. Proximal femoral endoprostheses (Figure 1), hip disarticulation, hindquarter amputation, and palliation were the treatment options. We analyzed patient, tumour, and treatment factors in relation to overall survival using Stat view software. Differences between groups were assessed using Mann Whitney *U*-test. Survival was estimated using Kaplan Meier survival curves with patients censored at the time of last followup. Significance was taken as *P* < 0.05.

## 3. Results

Between 1978 and 2008, seventeen patients had a pathological fracture of the proximal femur due to osteosarcoma. There were thirteen males and four females. Their age range was from 9 to 84 (mean age 42) with nine patients under the age of 40 and eight above the age of 40. (Table 1) Twelve patients had a fracture prior to diagnosis and five developed a fracture after the diagnosis. Seven patients (41%) had metastatic disease at diagnosis. Five patients were referred after fixation of the fracture prior to the diagnosis being made (a sliding hip screw fixation in four and intramedullary nail in one) of whom two had metastatic disease.

Two groups of patients were identified, those under the age of 40 and those over that age. (Table 1) This age was chosen as all of the younger group received chemotherapy and were offered surgery, whilst the older age group were usually only treated surgically or by palliation.

In the younger group of patients (age range 9 to 39), seven patients suffered fractures at the time of diagnosis and two later (one turning in bed and one stumbling on stairs). One patient had undergone nailing of a subtrochanteric fracture before the diagnosis had been made. Three patients had metastases at diagnosis. All were treated with immobilization and traction for the fracture, whilst they received neoadjuvant chemotherapy. Following restaging after chemotherapy, four patients were treated with endoprosthetic replacement of the proximal femur and four with amputation (two hip disarticulation and two hindquarter amputation). One patient developed progressive disease with metastases whilst on chemotherapy and declined amputation, receiving palliative radiotherapy.

The margins of excision were judged to be wide in three patients, marginal in three, and intralesional in two. Only two patients (both of whom had limb salvage) were found to have a good response to chemotherapy (>90% necrosis). Postoperative radiotherapy was given to patients with intralesional margins.

**Figure 2:**
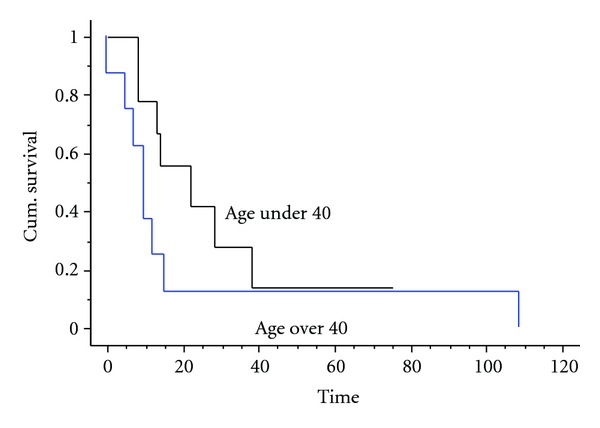
Kaplan Meier survival curve showing the survival of patients split by age group. There is no statistical difference between the two groups, despite the younger group all being treated with chemotherapy (*P* = 0.17).

All but one of this group of patients under 40 developed metastases (three had them at the time of diagnosis and five others developed them at 6, 7, 9, 19, and 21 months resp., following diagnosis). The only survivor in this group was the one without metastasis—patient 8 (Table 1). This patient had presented with a fractured proximal femur, had it nailed, but then the diagnosis of osteosarcoma was confirmed. He had no metastases at diagnosis and had progressive disease on chemotherapy leading to the need for hindquarter amputation. He remains disease-free after 75 months. The median survival of this group of young patients was 19 months with a one-year survival of 78%, two-year survival of 42%, and five-year survival of 14% (Figure 2).

The older group of nine patients were all over the age of 40, the youngest being 57 and the oldest 84 (Table 1). Three of the patients had underlying Paget's disease and five had conventional osteosarcoma. Four of the eight had lung metastases at the time of diagnosis (two of the three with a Paget's associated osteosarcoma) and four had undergone previous internal fixation for the fracture in the mistaken belief that it was due to metastatic disease.

**Figure 3:**
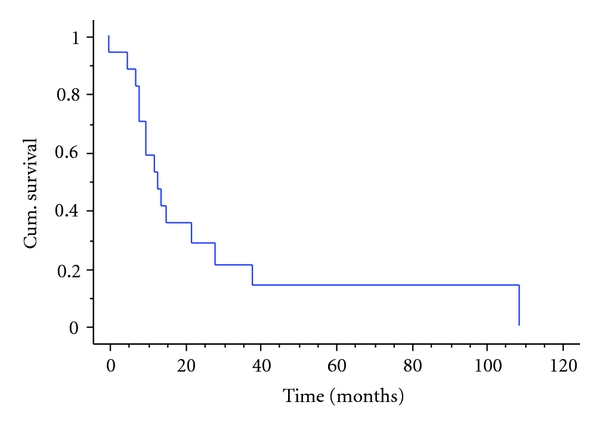
Kaplan Meier Survival analysis—osteosarcoma of the proximal femur with a pathological fracture—14% five-years survival.

Only two of these patients had chemotherapy, and this proved ineffective in both patients (20% necrosis in one and 25% in the other). Six of the eight underwent surgery with two having primary amputation and four limb salvage with an endoprosthesis. All of the patients eventually developed metastatic disease, with the four who did not have metastases at diagnosis developing them at 5, 6, 15, and 82 months following diagnosis. All of these patients have since died of their disease, the median survival being 9 months, the one-year survival 37%, and five-year survival 12% (Figure 2). The overall survival of the whole group was 14% at five years (Figure 3).

Eight patients had limb salvage surgery with a proximal femoral endoprosthesis, six patients underwent an amputation (three hindquarter and three disarticulation), and three had palliative treatment. Four patients had a local recurrence—three of the eight (37.5%) who had limb salvage surgery and one after hip disarticulation. There was no difference in survival between patients treated with amputation or limb salvage nor was there any difference in survival between those with metastases at diagnosis and those without, nor between those with fractures prior to diagnosis or after diagnosis. Of the five patients who had previous internal fixation of the fracture, one underwent primary amputation, three underwent limb salvage surgery, and one had palliative treatment. The only survivor is one who had a hindquarter amputation.

**Figure 4:**
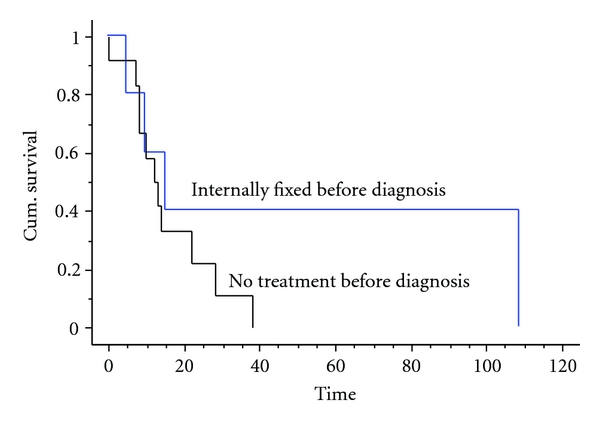
Kaplan Meier survival curve showing the survival of patients split by early fixation prior to diagnosis or no previous fixation. There was no statistical difference between the two groups (*P* = 0.19).

Two of the patients had intracapsular fractures, five had intertrochanteric fracture and ten had a subtrochanteric fracture. Again there was no difference in survival between them nor was there any difference between those with previous fixation of the fracture and those without (Figure 4).

Throughout the same time period of this study, the overall five-year survival for all patients with nonmetastatic osteosarcoma at our centre was 54% and for patients within this group with proximal femoral osteosarcoma but without a fracture was 51%.

## 4. Discussion

We have identified a group of patients with osteosarcoma who appear to have a very poor prognosis. Patients with a pathological fracture due to osteosarcoma of the proximal femur represent just 1.4% of all patients with osteosarcoma in our dataset, and if only patients under the age of 40 are included (as in most studies of osteosarcoma) then the proportion falls to under 1%.

Previous studies of osteosarcoma have identified a proximal location as having a poor prognosis and other studies have suggested that a pathological fracture offers a poor prognosis but the combination of the two has not previously been shown to be such a dismal combination. Most studies [1, 3, 5, 11–14] simply do not have enough patients to identify a group as small as this. Similarly, osteosarcoma in patients over the age of 40 has rarely been investigated with one large study only having 34 patients with osteosarcoma of the proximal femur, but no comment was made about their survival or the significance of a pathological fracture [1].

The reason why patients with a proximal femoral fracture do so badly is not easy to explain when they seem to do so much worse than patients with tumours at the same location without fractures.

Firstly, seven of this group of seventeen patients already had metastases at diagnosis (41%), a much higher proportion than is normally seen in osteosarcoma patients (11% at our institution). As these metastases will have seeded many months before the fracture happened, this might suggest an increased vulnerability of the location to metastasise and this would need verification in further studies.

Secondly, the risk of pathological fracture (31%) at this site is considerably higher than for any other location in the body, but this is probably simply a reflection on the local anatomy whereby any process that weakens the bone (e.g., osteoporosis) leads to an increased risk of proximal femoral fracture. The most common type of fracture was a subtrochanteric fracture, suggesting that most of the osteosarcomas arose in this region. Subcapital fractures were rare but affected management as the fracture haematoma was thus intracapsular. In that situation, the option of carrying out an extraarticular resection as described by Rüdiger et al. [2] could be considered although none of the patients in this series were treated in this way.

The main problem with proximal femoral fractures due to osteosarcoma is the lack of containment of the fracture haematoma by surrounding soft tissues. There is no enveloping muscle to prevent widespread local dissemination of haematoma and tumour cells, which is likely to track around all of the surrounding area including psoas, the glutei, adductors, and vasti. This means that any attempt at subsequent excision may have compromised margins with an increased risk of local recurrence. Five of our patients had undergone internal fixation of the fracture prior to the diagnosis being made, and this resulted in further extensive contamination of both bone and soft tissues with tumour cells.

The management of this group of patients is difficult. The normal management of a pathological fracture in osteosarcoma is to immobilize the fracture and administer chemotherapy in the hope that the fracture will heal and the fracture/tumour haematoma will become walled off, thus allowing limb salvage [3]. In the proximal femur, immobilization is difficult if not impossible either with traction or bed rest alone and can probably only be improved by application of a hip spica plaster or external fixation from the pelvis to the femur. Thus it is likely that in most cases there will be continuing movement at the fracture site, and this may offer an explanation for the high incidence of subsequent development of metastases in sixteen patients. If the fracture is internally fixed, then this does allow the patient to be mobile while treatment is considered, but on the other hand it will result in more widespread contamination of normal tissues and will then require even more extensive surgery to ensure clear margins can be obtained. In our five cases who had early stabilisation, one required an amputation and three had limb salvage of whom two developed a local recurrence. It would seem, therefore, that amputation is probably the only safe option following inadvertent fixation of a proximal femoral fracture in osteosarcoma unless there is a good response to chemotherapy. The only two patients in this series to survive more than five years had both undergone fixation of the fracture prior to diagnosis and both subsequently had amputation (one following attempted limb salvage and local recurrence).

Although there have been numerous other papers written about pathological fractures in osteosarcoma, the numbers with proximal femoral fractures were small and most papers make no comment about them compared to other sites.

Scully et al. [4] in 2006 reported a 30% risk of local recurrence and 60% two-year survival with limb salvage and 66% two-year survival with early amputation in a cohort of 16 patients with pathological fractures in non metastatic osteosarcoma (the group included four patients with a proximal femoral lesion). They did not find any statistically significant difference in survival between early amputation and limb salvage though there was 30% risk of local recurrence with limb salvage.

Scully et al. [5] in 2002 reported a multicentre case-matched retrospective study comparing fifty-two patients with a pathological fracture with fifty-five patients without a pathological fracture. The group included patients with non metastatic osteosarcoma, and the overall five-year survival was 55%. The cohort included seven patients with a proximal femoral lesion. They also noted that three out of five patients who had open reduction and internal fixation followed by limb salvage surgery were alive at an average of 6.1 years postoperatively.

Bacci et al. [3] reported overall survival of 65% from a cohort of 46 patients with nonmetastatic osteosarcoma of the extremities of whom 22 had involvement of the femur, but the number of proximal femoral lesions was not specified.

Given the rarity of this combination of proximal femoral fracture in osteosarcoma, providing evidence-based guidelines is likely to be difficult but based on our results thus far we suggest the following.

We believe that a pathological fracture in osteosarcoma of the proximal femur should be avoided at all cost. If there is an extensive lytic process likely to lead to fracture following biopsy, very early surgery should be considered, possibly doing a frozen section and being prepared to do a proximal femoral replacement prior to chemotherapy if the lesion is operable. The availability of modular endoprostheses [6] and the fact that chemotherapy is just as effective if administered postoperatively as preoperatively [7] may prevent the catastrophic complication of fracture.

If the fracture has already arisen and been inadvertently stabilised, then the patient should be treated conventionally with chemotherapy and reassessed. Limb salvage should only be considered if there has been a good response to chemotherapy. Amputation is likely to be the safest treatment option.

The most difficult group are those with an unstable fracture. If chemotherapy is not an option then amputation should be considered following diagnosis. If chemotherapy is being used (under the age of 60), then it should only be given preoperatively if the fracture can be completely immobilized and a good response is likely to lead to limb salvage. If this cannot be achieved then early amputation prior to chemotherapy should be considered. If the fracture can be simply immobilized by minimally invasive surgery, then this may possibly allow the administration of chemotherapy. Clearly these suggestions will need adapting for the individual, and it may be that in an older patient with metastases and limited life expectancy, early fixation or an endoprosthetic replacement may be all that is needed.

Accrual of multicentre data about proximal femoral pathological fracture due to osteosarcoma may shed further light into the management of this difficult problem to asses the potential benefits of early stabilisation or early radical surgery.

## Figures and Tables

**Table 1:** Patient, treatment and outcome details.

No.	Age	Type	Duration symptom in weeks	Path fract	Previous treatment	Site of fracture	Surgery	Chemo protocol	Chemo necrosis	Local recurrence	Mets	Time alive	Status
1	9 M	os	12	After diag	none	subtroch	amp	adria/cisplat	80	no LR	7	8	dead
2	12 F	os	8	At time diagnosis	none	subtroch	amp	adria/cisplat	30	no LR	6	13	dead
3	13 F	os	32	After diag	none	intracap	lss	pam	100	LR 20 months Rx RT	At diag	38	dead
4	14 M	os	8	After diag	none	subtroch	lss	pam	94	no LR	At diag	26	dead
5	17 F	os	12	At time diagnosis	none	subtroch	lss	adria/cisplat	80	no LR	19	22	dead
6	20 M	os	12	At time diagnosis	none	subtroch	amp	vcr/mtx		no LR	At diag	8	dead
7	26 M	os	12	At time diagnosis	none	intertroch		pam	0	no LR	9	14	dead
8	32 M	os	100	At time diagnosis	int fix	subtroch	amp	pam	25	no LR		75	alive
9	39 M	os	8	At time diagnosis	none	intracap	lss RT	adria/cisplat	20	no LR	21	28	dead
10	57 M	pagets	25	After diag	none	subtroch	amp	no		no LR	6	12	dead
11	62 F	os		After diagn	int fix	intertroch	lss	no		no LR	At diag	5	dead
12	62 F	os	26	At time diagnosis	none	intertroch	lss RT	no		no LR	5	7	dead
13	65 M	os	12	At time diagnosis	int fix	subtroch	lss	adria		LR 15 months Rx Amp	82	109	dead
14	67 M	os	26	At time diagnosis	int fix	intertroch	lss	no		LR 4 months Rx Excise and RT	15	15	dead
15	67 M	pagets	104	At time diagnosis	none	subtroch	amp	no		no LR	At diag	10	dead
16	74 M	os	40	At time diagnosis	int fix	intertroch		no		no	At diag	10	dead
17	84 M	pagets	36	At time diagnosis	none	subtroch		no		no	At diag	1	dead
